# Editorial: Novel applications of ONT technologies in genomics and transcriptomics

**DOI:** 10.3389/fgene.2024.1384584

**Published:** 2024-03-20

**Authors:** Eugenia Poliakov, Ludmila Kaplun, Igor B. Rogozin

**Affiliations:** ^1^ Laboratory of Retinal Cell and Molecular Biology, National Eye Institute (NEI), National Institutes of Health, Bethesda, MD, United States; ^2^ Variantyx Inc., Framingham, MA, United States; ^3^ Life Science Research Center, Faculty of Science, University of Ostrava, Ostrava, Czechia

**Keywords:** ONT long read sequencing, ONT full-length transcriptome sequencing, long read DNA sequencing, genomic assays, ONT

PacBio and Oxford Nanopore sequencing technologies have been developed rapidly in the last decade. The Oxford Nanopore sequencing is based on a direct measurement of the electric current that flows through the nanopore embedded in an electro-resistant membrane. It eliminates a need for PCR-based amplification of DNA/RNA and allows the identification of epigenetic modifications. This rapid pace of ONT technological revolution can be seen worldwide especially for low-cost rapid nucleotide sequencing. ONT markets have developed rapidly in the past decade. Four generations of nanomaterials have been developed. The quality (raw sequencing error rate and the length of reads) of ONT has being improved literally each month and now is in excess of 99% for direct sequencing chemistry. The Research Topic attracts many downloads and receives many citations, but as the technology is rapidly evolving, regular updates on this technology are required.

Why ONT has become so popular? The major ONT advantage is a possibility to effectively sequence very long reads that could be up to hundreds of thousands of nucleotides while simultaneously detecting native epigenetic modifications. Analysis of ONT data is not standard yet due to complexity of bioinformatics pipelines that allow for analyses of long reads that contain a higher rate of sequencing errors compared to the short-read technologies. However, many applications of ONT data analysis are relatively straightforward and easy to apply. A comprehensive and interactive catalogue of analysis tools for long-read sequencing data is available at the “long-read-tools.org” website (https://long-read-tools.org/index.html). This is an open-source centralized database that allows exploration of long-read data analysis tools through interactive browsing and filtering. Currently, the database contains more than 500 tools across 32 categories of data analyses. The most frequent analysis tasks include base calling, *de novo* assembly, error correction, quality controls and filtering, while long-read single-cell data analysis and transcriptomics (including isoform detection) are areas with the fewest tools available at the “long-read-tools.org” website.

This Research Topic begins with a comprehensive review “Resolving complex structural variants via nanopore sequencing” by Romagnoli et al. which discussed the successful applications of ONT approach for recent developments of NGS (next-generation sequencing) platforms for detection of structural variants (SVs) as the hallmark of genome instability, potentially leading to pathologic conditions. ONT long-reads and its combination with the short reads have already been proven to be invaluable in overcoming limitations of short-read sequencing to resolve wide and structurally complex SVs (Mahmoud et al., 2019; Cleal and Baird, 2022; Greer et al., 2023). This review also discusses bioinformatics methods that improve the identification of SVs associated with human pathological conditions, discussing the possibility of introducing nanopore sequencing technology into routine clinical diagnostics. Overall, this review clearly shows the promises and pitfalls of ONT application to diagnostics. This paper is accompanied by three research papers that cover different fields of ONT applications.

Kaplun et al. “ONT long-read WGS for variant discovery and orthogonal confirmation of short read WGS derived genetic variants in clinical genetic testing,” shows how it is possible to improve quality of variant detection using ONT. In fact, the paper is a logical research continuation of the previously discussed paper Romagnoli et al. The authors have developed an integrated clinical genetic testing approach, augmenting short read WGS-based variant detection with long read sequencing, providing simultaneous orthogonal confirmation of all types of variants with the additional benefit of improved identification of exact size and position of the detected aberrations. The validation study of this augmented test has demonstrated that ONT sequencing can efficiently verify multiple types of reportable variants, thus ensuring highly reliable detection and a quick turnaround time for WGS-based clinical genetic testing.

The paper “Discovering novel reproductive genes in a non-model fly using *de novo* GridION transcriptomics” by Walter and Puniamoorthy demonstrates how high-thruput ONT can be used for gene discovery, with important implications for investigating phenotypic trait evolution, adaptation, and speciation. It was found that 80% of genes encoding secretory proteins account for 74% total gene expression. It is likely that rapid genomic innovation with recruitment of *de novo* genes for high expression in *S. punctum* accessory glands could be a likely mechanism of evolution of these genes. The study also demonstrates the feasibility of adapting ONT transcriptomics for gene discovery in non-model systems.

ONT can be applied to genomics as well. The paper “*De novo* assembly of a chromosome-level reference genome of the ornamental butterfly *Sericinus montelus* based on nanopore sequencing and Hi-C analysis” by Li et al. shows how ONT may be used for the whole genome reconstruction. This is certainly a fast-growing and prominent field of ONT applications. The authors demonstrate that approximately 48.86% of the assembled genome was suggested to be repeat elements that makes assembly of this genome using short reads an almost impossible task. The authors used short reads, ONT long reads and high-throughput chromosome conformation capture (Hi-C) analysis Kaplun et al. to identify genome-wide interactions between and within chromosomes. 13,720 protein-coding genes were predicted. The *de novo* assembly of a high-quality reference genome for *S. montelus* provided a fundamental genomic tool for future research on evolution, genome genetics, and adaptability to toxic plants of the swallowtail butterflies.

Despite all the progress, there are numerous ONT technical issues that are not completely resolved yet. The paper “Improved Nanopore full-length cDNA sequencing by PCR-suppression” by Bayega et al. demonstrates some shortcomings of widely used ONT SQK-PCB109 chemistry and ways that can improve this methodology. The authors suggested that previously developed technique that is based on addition of inverted terminal repeats in cDNA during reverse transcription followed by single-primer PCR creates a PCR suppression effect that prevents amplification of short molecules thus enriching the library for longer transcripts. The authors adapted this method for nanopore cDNA library preparation and show that not only is PCR efficiency increased but gene body coverage is dramatically improved. The results show that implementation of this simple strategy will result in better quality full-length RNA sequencing data and make full-length transcript sequencing possible for most sequenced reads. ONT sequencing requires a constant upgrading to library preparation and development of PCR-based methods.

Finally, the paper “Modification mapping by nanopore sequencing” by White and Hesselberth describes one of the most problematic tasks for the whole field of genomics/transcriptomics, namely, detection of modified nucleotides. The authors describe the latest achievements in the field while introducing the reader to nanopore sequencing and key principles underlying its use in direct detection of nucleic acid modifications in unamplified DNA or RNA samples. The paper outlines current approaches for detecting and quantifying nucleic acid modifications by Oxford nanopore sequencing. As this technology matures, the authors anticipate that advances in both sequencing chemistry and analysis methods will lead to rapid improvements in the identification and quantification of these epigenetic marks.

All in all, we think that this Research Topic is a useful compilation of high-quality papers by produced six groups of outstanding researchers that show many “hidden” details of ONT use. We hope that this overview of nanopore sequencing covers major advantages and problems of ONT.
